# Chronic Myeloid Leukemia: Chronic Phase Presenting as Extramedullary Presentation

**DOI:** 10.7759/cureus.32691

**Published:** 2022-12-19

**Authors:** Vedant A Nanote, Ojas A Mahajan, Shilpa A Gaidhane, Swapnil D Parve, Lalit Raut

**Affiliations:** 1 Department of Primary Care & General Practice, Kazan State Medical University, Kazan, RUS; 2 Department of Medicine, Jawaharlal Nehru Medical College, Datta Meghe Institute of Medical Sciences, Wardha, IND

**Keywords:** tyrosine kinase inhibitors (tkis) therapy, parotid gland, chronic phase, chronic myeloid leukemia, myeloid sarcoma

## Abstract

Chronic myeloid leukemia (CML) is a myeloproliferative disorder characterized by immature granulocytes in the peripheral blood and bone marrow. In 95% of cases, it is always associated with the presence of the Philadelphia chromosome, which is characterized by the presence of reciprocal translocation between chromosomes 9 and 22. However, 3.1-9.1% of patients also have an extramedullary proliferation of skin, lymph nodes, bone, or central nervous system (CNS), which could be either myeloid, lymphocytic, or mixed lineage in origin. An extramedullary myelogenous neoplasm termed myeloid sarcoma (MS) can originate from myeloblasts or immature myeloid cells. Due to the green, gross appearance caused by the myeloperoxidase enzyme in immature myeloid cells, it is also known as chloroma. According to WHO guidelines, it is a tumor composed of myeloid blasts, mature or immature. Here we report an old female patient with CML - chronic phase who came for imatinib therapy and presented as MS in the right parotid gland.

## Introduction

Myeloid sarcoma (MS) is an extramedullary tumor composed of immature myeloid cells that destroy the underlying tissue architecture. It is usually associated with acute myelogenous leukemia but can be associated with myeloproliferative neoplasms, myelodysplastic disorders, or myeloproliferative/myelodysplastic syndromes [1]. If undetected it may delay appropriate chemotherapy and jeopardize survival [2,3]. MS is a tumor mass that arises in anatomical sites other than the bone marrow [4]. MS mostly occurs during the accelerated phase or the blast phase in the bone marrow and peripheral blood but rarely in the chronic phase. However, we present a patient presenting with chronic myeloid leukemia (CML) - chronic phase having MS in the right parotid gland.

## Case presentation

Patient information

**Figure 1:**
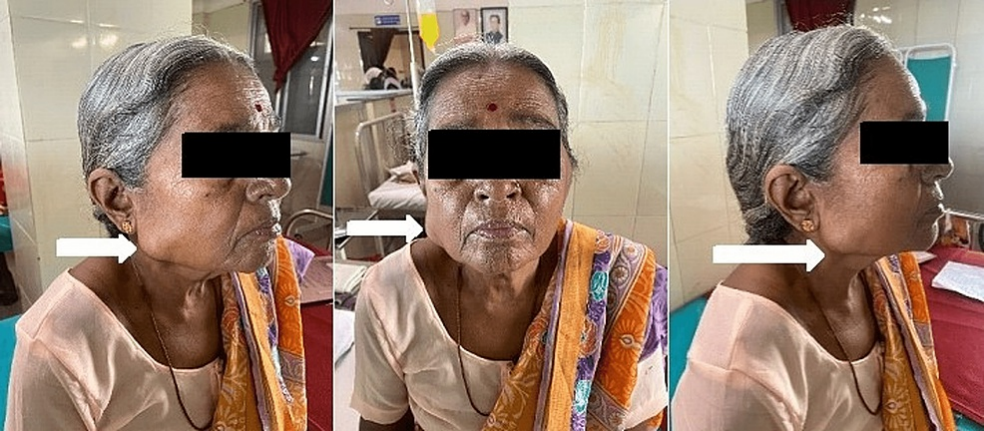
Patient presenting with chronic myeloid leukemia - chronic phase having mass around the right parotid gland

A 69-year-old female came to the outpatient medical clinic for swelling in the right parotid gland. She also complained of dyspnea on exertion and lassitude. A month ago, the patient seemed to be doing well when she observed a mass over her right parotid gland which was insidious in onset, initially not present, and gradually progressed to its present size. Physical examination revealed an enlarged cervical lymph node, parotid gland swelling, and splenomegaly. On palpation of the parotid gland, it revealed a 2x2 cm round, hard, painless, non-tender mass that was soldered to the parotid gland without skin and subcutaneous infiltration (Figure 1).

Past medical history

In 2019, she was diagnosed to have CML. Her peripheral smear showed normocytic erythrocytes cells with adequate platelets on smear, basophilia, leukocytosis with a total leukocyte count (TLC) of 2,00,000/cu mm (normal range - 4,000-11,000/cu mm), and differential leukocytes count of myeloblast 4%, promyelocytes 8%, myelocytes 21%, metamyelocytes 19%, band forms 8%, polymorphs 28%, and lymphocytes 6%. Bone marrow biopsy was suggestive of increased myeloid cells, erythroid cells with normoblastic erythropoiesis, and a few megakaryocytes with normal morphology. For her CML tab imatinib 400 mg twice daily was started and she was advised to do complete blood counts once in every month for monitoring. The patient is known hypertensive for 10 years on regular medications.

Current laboratory analysis

**Figure 2:**
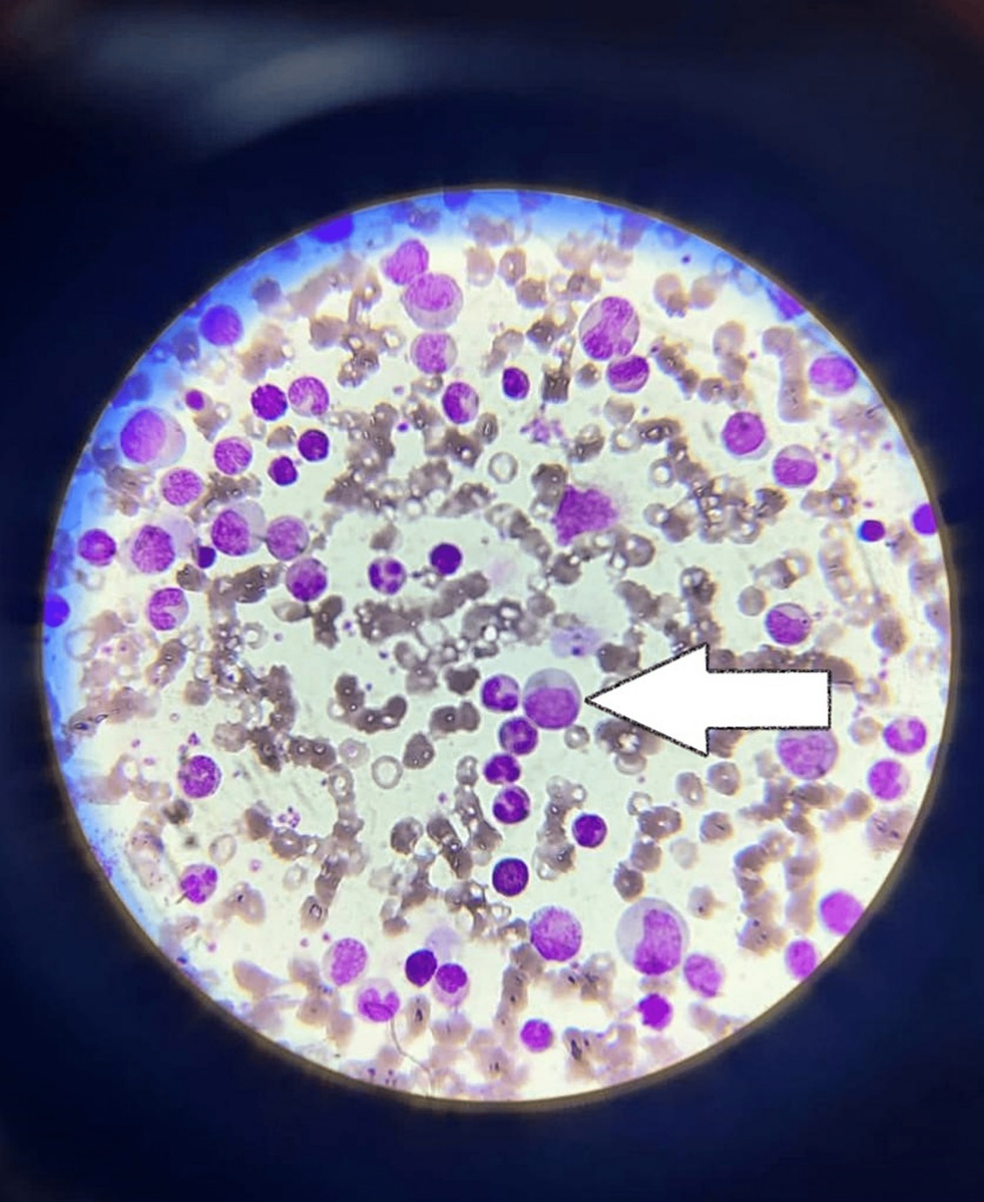
Peripheral smear of blood sample showing Myeloid Bulge

Her hemoglobin is 9 gm/dl and her TLC is 83,800 cells/cu mm. RBC mass appeared reduced on the peripheral smear. RBC precursors (intermediate and late normoblasts) are seen. Normocytic normochromic anemia with few microcytes. Platelets (2.13 cu mm) are adequate on the smear. Peripheral smear findings were suggestive of normocytic anemia, leukocytosis with ''Myelocyte Bulge'' with band forms, neutrophils, and other immature cells. Ultrasound's local site revealed a well-designed hypoechoic lesion measuring approximately 18x16 mm in the superficial lobe of the right parotid gland (Table 1) (Figure 2).

**Table 1:** Current differential leukocytes count

Cells	Results	Normal Range
Myeloblasts	05%	0-1.5%
Promyelocytes	10%	2.0-4.1%
Myelocytes	12%	8.2-16%
Metamyelocytes	12%	9.6-24.6%
Band forms	04%	9.5-15.3%
Lymphocytes	08%	30-50%
Monocytes	0%	2-8%
Eosinophils	03%	1-4%
Basophils	10%	0-1%

**Figure 3:**
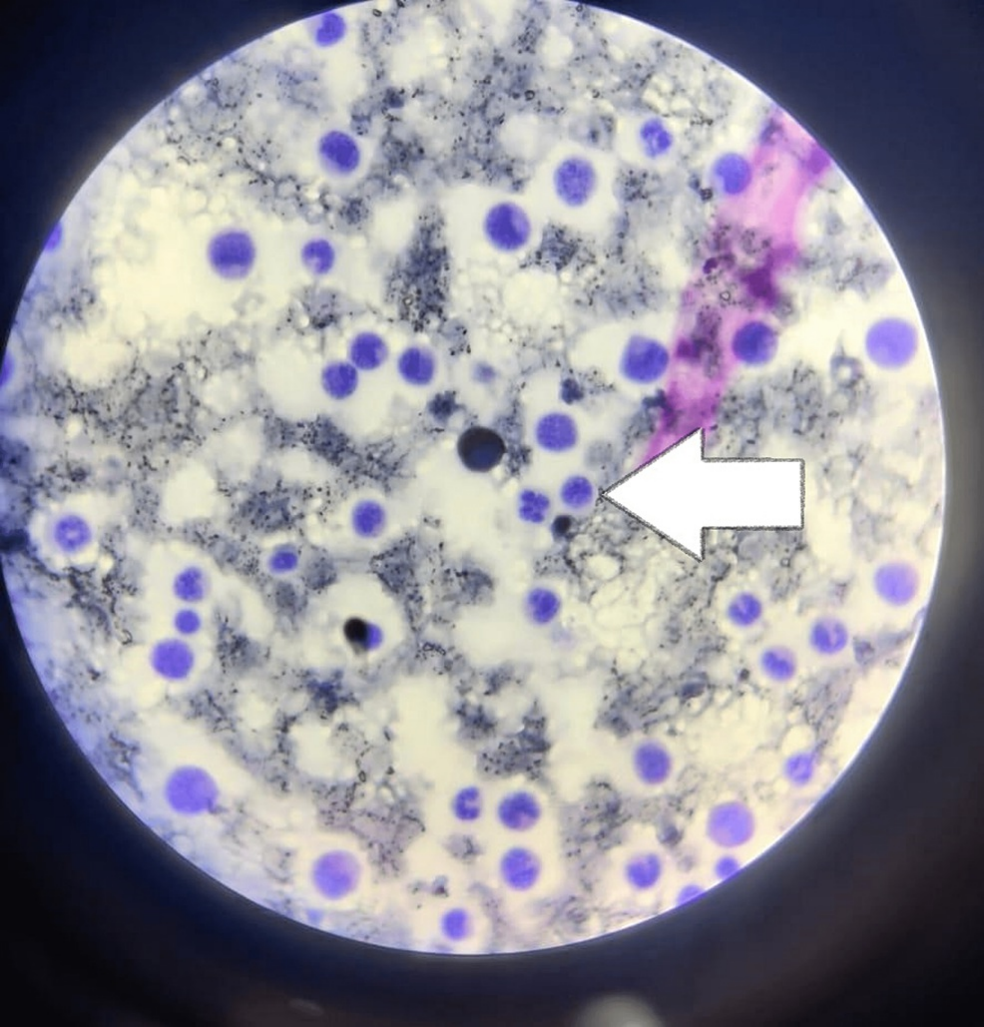
Fine needle aspiration cytology (FNAC) of mass over the right parotid gland showing myeloperoxidase positive cells

Fine needle aspiration cytology (FNAC) of parotid gland swelling was done and cells strongly expressed MPO (myeloperoxidase), a known myeloid cell marker, and showed infiltration of CML, and no parotid tumor was noticed. Under high magnification power, large neoplastic cells were observed in the lymphoid stroma of the parotid gland. The cells were round, discrete, and twice the size of mature lymphocytes. The nuclei were round to oval with fine chromatin and 2-3 nucleoli. As a result, the pathologic diagnosis was confirmed as MS in the parotid gland with preexisting CML (Figure 3).

Therapeutic interventions

Considering the patient's hypertensive comorbidity we prescribed her a combination of tab atenolol, amlodipine (50/5) mg once a day, and tab aspirin 75 mg once a day. For CML tab imatinib 400 mg twice daily and was advised to do complete blood counts once in every two weeks for monitoring. As the patient developed MS on imatinib therapy, the patient was switched to tab dasatinib 50 mg twice daily. The patient was advised quantitative reverse transcription-polymerase chain reaction (RT-PCR) (qPCR) for BCR-ABL1 but economically she is not much stable, hence she cannot afford the cost.

## Discussion

Our patient has a rare condition, an extramedullary myelogenous sarcoma in parotid during the chronic phase of CML. However, most CML patients present with extramedullary involvement during blast crises. Here, our patient has clinical laboratory results in the chronic phase but shows extramedullary parotid swelling and the FNAC report shows MPO-positive cells, indicating MC. The patient was shifted to tab dasatinib as she developed MC on imatinib therapy. Moreover, drug monitoring should be done to rule out imatinib resistance in a patient and second-line tyrosine kinase inhibitors should be prescribed such as dasatinib, bosutinib, nilotinib, or third-line: ponatinib. We can also try dose escalation but in a patient, we can see the suboptimal response to imatinib therapy. MC is commonly seen with myelodysplastic syndrome (MDS), myeloproliferative syndrome (MPS), polycythemia vera, and essential thrombocytosis. The most common solid organs involved in MC are the mediastinum, lymph nodes, and skin (leukemia cutis) [1]. It has also been reported that tumors have grown in the epidural space, uterus, ovaries, testicles, and orbits. Months prior to the involvement of bone marrow and peripheral blood, the soft tissue equivalents may start showing up. Depending on their anatomical location, they may cause symptoms, however, they can also be asymptomatic [1,2]. A biopsy is necessary for a definitive diagnosis. Collections of cells that may typically be identified as myeloid are visible under a light microscope. Undifferentiated or marginally differentiated blasts can cause diagnostic challenges, leading to cases wherein lymphoma is incorrectly diagnosed. Immunohistochemistry and cytochemistry must be used to aid in the diagnosis. Immunohistochemistry is the investigation of choice for diagnosing MC [1,2]. Monoclonal antibodies used in immunohistochemistry are MPO; anti-CD43, anti-lysozyme (most sensitive); CD68 and CD20 (to rule out lymphoma) [2]. Disease monitoring by complete blood counts is done once in every two weeks [3,4]. Bone marrow biopsies have to be done in the period of every six months to check the cytogenic responses. Once a bone marrow biopsy and cytogenic response are achieved quantitative RT-PCR (qPCR) for BCR-ABL1 should be done on peripheral blood at 3-6 month intervals [5]. If after monitoring the disease, the patient is diagnosed under accelerated phase or blast crisis it should be treated accordingly [6]. The accelerated phase is treated with high doses of cytarabine (HDAC) and hydroxyurea (Hydrea). Blasts crisis is treated like acute myeloid leukemia - the first step is induction chemotherapy, we may follow regimes such as DA 3+10 - Daunorubicin 50mg/m^2^ on days 1, 3, 5, and cytarabine 100mg/m^2^ twice a day on days 1-10. DA 3+8 - Daunorubicin 50mg/m^2^ days 1, 3, 5, and cytarabine 100mg/m^2^ twice a day on days 1-8. The second step is consolidation chemotherapy we may follow regimes such as MACE - Etoposide 100mg/m^2^ on days 1-5, Amsacrine 100mg/m^2^ on days 1-5, Cytarabine 200mg/m^2^ on days 1-5 [6,7].

## Conclusions

This is a unique case as there is extramedullary presentation i.e. MS in the parotid gland in the CML in the chronic phase. Managing this situation requires timely monitoring, optimal dose of drugs, and good adherence, which is challenging for the developing rural population of India due to financial and economic challenges.
